# Interactions Between Root Traits and Fungal Functional Guilds Across the Root Economics Spectrum

**DOI:** 10.3390/plants15071031

**Published:** 2026-03-27

**Authors:** Xinyi Chen, Jie Zhang, Zhirong Liu, Jian Guo, Yaoyao Tong, Qiu Yang, Guilong Li, Jia Liu

**Affiliations:** 1 Institute of Soil and Fertilizer & Resources and Environment, Jiangxi Academy of Agricultural Sciences, Nanchang 330200, China; ppchen0701@163.com (X.C.); n1727640389@163.com (Q.Y.); glli@jxaas.cn (G.L.); 2 School of Soil and Water Conservation, Jiangxi University of Water Resources and Electric Power, Nanchang 330099, China; lzr6270@163.com (Z.L.); ggjian2016@163.com (J.G.); tongyaoyao2023@163.com (Y.T.); 3 National Engineering and Technology Research Center for Red Soil Improvement, Nanchang 330200, China; 4 Key Laboratory of Acidified Soil Amelioration and Utilization, Ministry of Agriculture and Rural Affairs, Nanchang 330200, China; 5 Key Laboratory of Crop Physiology, Ecology and Farming in the Middle and Lower Reaches of the Yangtze River, Ministry of Agriculture and Rural Affairs, Nanchang 330200, China; 6 Jiangxi Province Key Laboratory of Arable Land Improvement and Quality Enhancement, Nanchang 330200, China

**Keywords:** root economics space, green manure, root functional strategy, fungal guilds, functional prediction

## Abstract

Soil fungi play a pivotal role in maintaining ecosystem functions and regulating plant health. Although plant root traits can significantly impact the abundance and diversity of different fungal groups, the mechanism by which plant root strategies drive the assembly of soil fungal guilds remains limited. Utilizing Root Economics Space theory, this study investigates how four green manures (hairy vetch, rye, radish, and rapeseed) with contrasting root functional strategies (along the ‘fast–slow’ and ‘outsourcing–DIY’ axes) regulate the composition and functional structure of soil fungal communities. Community characteristics of three functional guilds (plant pathogens, saprophytes, and arbuscular mycorrhizal fungi), as well as relationships between these communities and plant root traits, were evaluated using a combination of Illumina high-throughput sequencing, functional annotation, and multivariate statistical analysis. Overall, different root strategies were associated with distinct fungal community patterns, potentially related to differences in root-derived resource inputs and soil properties. The ‘slow’ and ‘DIY’ strategies were associated with lower relative abundance of plant pathogenic fungi and higher relative abundance of saprotrophic fungi, whereas the ‘fast’ and ‘outsourcing’ strategies were associated with higher relative abundance of plant pathogens and AMF. These findings suggest that root functional strategies may help explain variation in fungal guild composition under different green manure species. From a practical perspective, the results provide a basis for selecting green manure species to help manage soil-borne disease risk, regulate beneficial soil microbial communities, and support more sustainable soil management in agricultural production.

## 1. Introduction

As either symbionts (e.g., arbuscular mycorrhizal fungi [AMF]) or antagonists (e.g., plant pathogens and saprophytes), soil fungi play a dual role in regulating plant growth and ecosystem functions [1]. As such, identifying the key factors influencing fungal guild composition is crucial for understanding the mechanisms underlying plant-microbe interactions as well as for promoting soil health and sustainable management practices [2]. Serving as a link between plants and soil microbiota, plant root traits exert significant selective pressures on soil fungal communities [3]. However, trait-based ecological research still primarily focuses on aboveground traits, and comparatively little is known about the ecological consequences of belowground traits, particularly root strategies.

Even so, trait-based research frameworks have continued to expand, including the development of the Root Economics Space (RES) proposed by Joana Bergmann [4]. This plant economic spectrum theory encompasses two orthogonal axes of root trait variation: the ‘fast-slow’ axis (i.e., conservation gradient) and the ‘outsourcing/do-it-yourself’ (hereafter ‘DIY’) strategy (i.e., collaboration gradient) [4,5,6]. Oscar J. Valverde-Barrantes empirically validated RES theory by studying variation in tree root traits, effectively differentiating between the collaboration and conservation axes [7]. The concept of the Root Economics Spectrum has stimulated research on the correlations between RES and plant traits, including the relationship between Leaf Economics Spectrum (LES) and RES [3], as well as the feedback mechanisms of root traits on root exudates [8]. For example, root traits, particularly those associated with RES, have been shown to directly influence root exudates and indirectly affect the functional assembly of fungal communities [4], thereby establishing a connection between root trait variation and soil-borne fungi.

Both the diversity and functions of soil fungi are jointly influenced by soil physicochemical properties and plant-related traits. Soil fungal communities exhibit high functional diversity and are typically classified into guilds based on their specific ecological niches, including plant pathogens, saprophytes, and AMF, among others [9]. Different fungal guilds interact with plant roots in distinct ways. For example, AMF promote ecosystem stability by promoting plant nutrient uptake and enhancing stress resistance [10,11], pathogens increase the risk of plant disease in carbon-rich or acidic environments [12], and saprophytes influence the carbon cycle by decomposing leaf litter and other organic debris [13]. On the other hand, fungal abundance and diversity can be influenced by a variety of factors, including the addition of utilizable resources [1,14,15]. Shifts in community structure and function reflect differences in resource utilization strategies and niche differentiation processes among fungal guilds, and are often driven, at least in part, by interspecific interactions [16]. Therefore, exploring how plant root strategies influence the structure and function of soil fungal guilds may shed light on plant–microbe interactions and their ecological effects.

In agricultural production, green manures play a vital role in regulating soil health and sustainably enhancing crop productivity [17,18]. Notably, variation in green manure root traits is recognized as a key factor influencing the evolution of soil microbial communities [19,20]. Specifically, the use of green manure has been found to enrich specific fungal taxa by regulating soil pH [21,22], increasing organic matter content [23], and modifying crop root exudates [24]. Furthermore, green manure can influence the prevalence of plant pathogens and the persistence and function of beneficial fungi such as AMF [25,26]. However, the majority of research into the impact of green manure on microbial communities has primarily focused on the effects of crop species and interplanting [27,28,29], without thoroughly investigating how the root functional strategies of these crops influence soil fungal guilds. Given the critical role of root traits in regulating fungal niches, analyzing green manure utilization through the RES lens is expected to clarify the effects of different green manure strategies on fungal guild structures, thereby enhancing our understanding of plant-microbe interactions.

Based on this framework, the present study examined four green manure species with contrasting root functional strategies. We hypothesized that green manures differing in root strategies would be associated with distinct patterns in soil fungal guild composition and diversity. Specifically, we expected that ‘fast’ and ‘outsourcing’ strategies would be associated with higher relative abundances of plant pathogens and AMF, whereas ‘slow’ and ‘DIY’ strategies would be associated with higher relative abundances of saprophytic fungi. Therefore, the objective of this study was to evaluate how green manure root strategies relate to the composition and functional structure of soil fungal guilds, and to provide a theoretical basis for green manure selection and soil microbial management in agricultural systems.

## 2. Results

### 2.1. Differentiation of the Economic Space of Green Manure Root Systems

**Figure 1:**
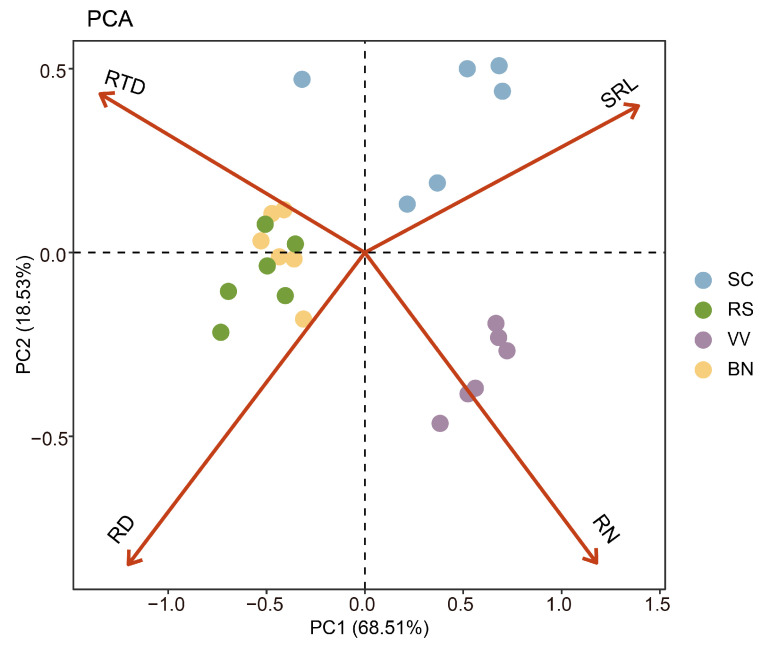
Principal component analysis (PCA) of green manure root traits. Principle component 1 (PC1) explained 68.51% of the variance and PC2 explained 18.53% of the variance. Scattered points represent the distribution characteristics of different treatments. VV, hairy vetch; RS, radish; BN, rapeseed; SC, rye. RD, root diameter; RN, root N content; RTD, root tissue density (root dry mass: root volume); SRL, specific root length (root length: dry mass).

PCA was conducted to thoroughly evaluate the root system characteristics of various green manures. Overall, these traits demonstrated a significant degree of similarity within the RES of the biological community and two distinct PC axes were observed (Figure 1). Accounting for 68.51% of the variation, PC1 illustrates the cooperation gradient, ranging from larger RD (‘DIY’ strategy) to higher SRL (‘outsourcing’ strategy). Regarding green manures, the RS and BN treatments were found to embody the DIY strategy while the VV and SC treatments can be categorized as possessing an outsourcing strategy. PC2, which accounted for 18.53% of the variation, represented a conservation gradient from high RTD (indicative of ‘slow’ roots) to high RN (characteristic of ‘fast’ roots). Crop roots under the VV treatment exhibited a typical ‘fast’ strategy within this dimension, characterized by elevated N content. Conversely, root systems associated with RS, BN, and SC treatments displayed a ‘slow’ strategy, primarily characterized by higher RTD.

Although a higher RD typically represents an ‘outsourcing’ strategy, this classification is not absolute because the RES is a continuous multidimensional space in which the positions of different species along specific axes are flexible. Diverse strategy combinations among species may result in the positioning of root traits along PC axes misaligned with traditional assumptions. For example, we observed that the two tap rooted species, radish and rapeseed, exhibited a higher RD primarily due to morphological characteristics while their lower SRL aligned with their ‘DIY’ strategy. As such, the positioning of radish and rapeseed at the high end of RD, while displaying a ‘DIY’ strategy, does not violate the core logic of the RES framework.

### 2.2. Association of Fungal Functional Groups with Environmental Factors

**Figure 2:**
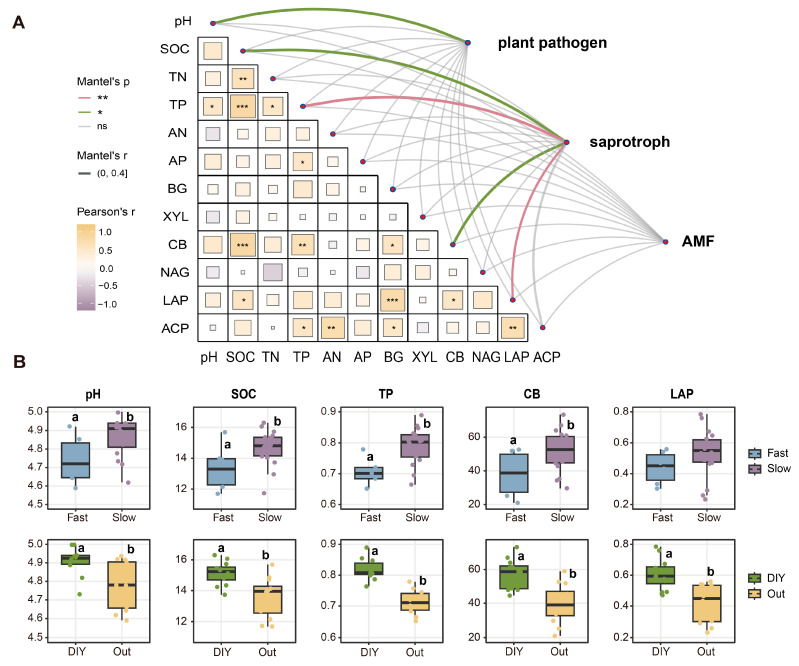
Relationships between soil properties, fungal guilds, and root traits. (**A**) Correlations between environmental variables and fungal guilds based on the FungalTrait database. Correlations were analyzed via partial Mantel tests, with Mantel R statistics ranging from 0 to 0.4. The green and pink lines represent statistical significance based on 999 permutations, whereas the gray lines indicate nonsignificant correlations (ns). The gray lines indicate nonsignificant correlations. Pairwise comparisons are displayed in a triangular heatmap, with correlation types represented by color gradients and significant correlations denoted by symbols (* *p* < 0.05; ** *p* < 0.01; *** *p* < 0.001). (**B**) Relationships between soil physicochemical properties and root traits. AN, available nitrogen; TN, total nitrogen; SOC, soil organic carbon; AP, available phosphorus; TP, total phosphorus. BG, β-1,4glucosidase activity; XYL, β-xylosidase activity; CB, β-D-cellobiohydrolase activity; NAG, β-1,4-N-acetylglucosaminidase activity; LAP, leucine aminopeptidase activity; ACP, acid phosphatase activity; AMF, arbuscular mycorrhizal fungi; DIY, do-it-yourself strategy; Out, outsourcing strategy. Different lowercase letters above the boxes indicate significant difference among the treatments (*p* < 0.05).

Soil environmental factors are significant drivers of soil microbial community structure and function. Based on the functional type outlined in the FungalTrait database, we identified three main fungal functional groups: plant pathogens, saprotrophs, and AMF. Notably, we observed a close association between the abundance of saprotrophs and SOC, TP, CB activity, and LAP activity (*p* < 0.05), while pH primarily impacted plant pathogens (*p* < 0.05) (Figure 2A).

Subsequently, we analyzed the effects of various root traits on pH, SOC, TP, CB, and LAP activity. Our results indicated that the pH, SOC, TP, and CB of green manures exhibiting ‘slow’ root traits or employing ‘DIY’ root strategies were significantly higher than those of green manures with ‘fast’ root traits or ‘outsourcing’ root strategies (*p* < 0.05) (Figure 2B). Notably, LAP activity exhibited significant differences solely in the comparison between ‘outsourcing’ and ‘DIY’ strategies (Figure 2B).

### 2.3. Responses of Fungal Functional Groups to Different Root Trait Strategies

**Figure 3:**
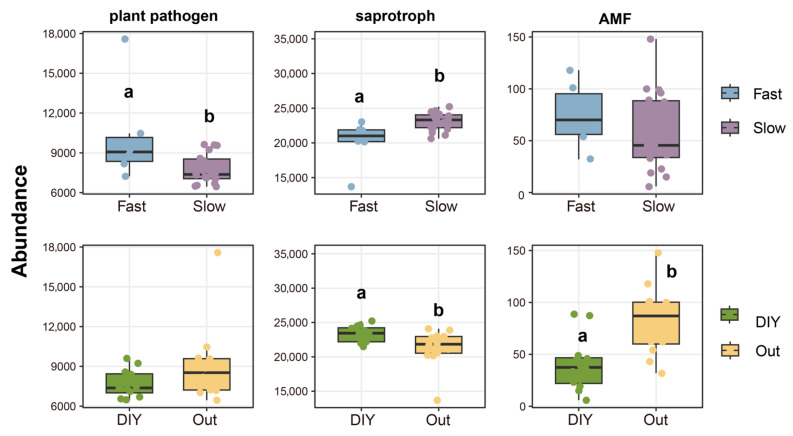
The effects of different root strategies on the abundances of plant pathogenic fungi, saprophytic fungi, and arbuscular mycorrhizal fungi (AMF). Different letters represent statistically significant differences (*p* < 0.05). DIY, do-it-yourself strategy; Out, outsourcing strategy.

Next, we analyzed the relationships between green manure root traits and the relative abundances of plant pathogens, saprophytes, and AMF (Figure 3). Plant pathogenic fungi were significantly more abundant on green manures exhibiting ‘fast’ root traits compared to those with ‘slow’ root traits (*p* < 0.05) (Figure 3). Conversely, saprophytic fungi were significantly less abundant on green manures with ‘fast’ root traits and significantly more abundant on those with ‘DIY’ traits. AMF abundance differed significantly between green manures employing an ‘outsourcing’ strategy and those utilizing a ‘DIY’ strategy (*p* < 0.05) (Figure 3). However, no significant differences were observed between those with ‘fast’ or ‘slow’ root traits. Together, these results indicate that saprophytes were relatively enriched under ‘slow’ roots with ‘DIY’ strategies, whereas plant pathogens and AMF were relatively enriched under ‘fast’ roots with ‘outsourcing’ strategies.

**Figure 4:**
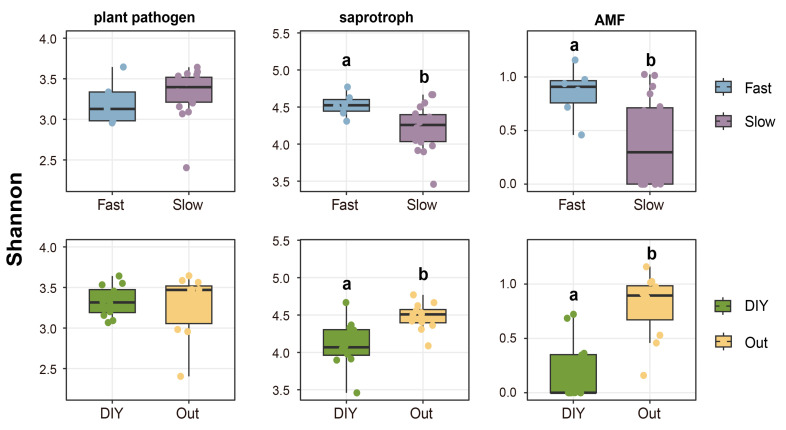
The effects of different root strategies on the α-diversities (Shannon indices) of plant pathogenic fungi, saprophytic fungi, and arbuscular mycorrhizal fungi (AMF). Different letters represent statistically significant differences (*p* < 0.05). DIY, do-it-yourself strategy; Out, outsourcing strategy.

**Figure 5:**
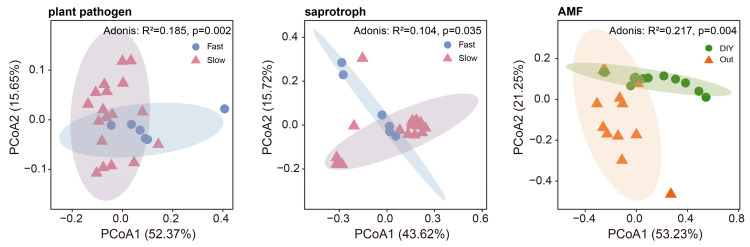
The effects of different root strategies on the β-diversities of plant pathogenic fungi, saprophytic fungi, and arbuscular mycorrhizal fungi (AMF). Ellipses of different colors represent the 95% confidence intervals (CI) for different root system traits within the principal coordinates analysis (PCoA).

We further analyzed the α- and β-diversities of fungal communities associated with different root traits. The Shannon indices of saprophytic fungi (Figure 4) and AMF (Figure 4) were significantly higher (*p* < 0.05) on ‘fast’ green manures roots employing ‘outsourcing’ strategies. According to the PCoA of β-diversity, PCoA1 and PCoA2 explained a combined 68.02%, 59.34%, and 74.48% of variation in the pathogen, saprophyte, and AMF communities, respectively (Figure 5). The adonis analysis indicated that observed differences in community structure were statistically significant (*p* < 0.05).

### 2.4. Differentially Enriched Fungal Taxa Associated with Different Root Trait Strategies

**Figure 6:**
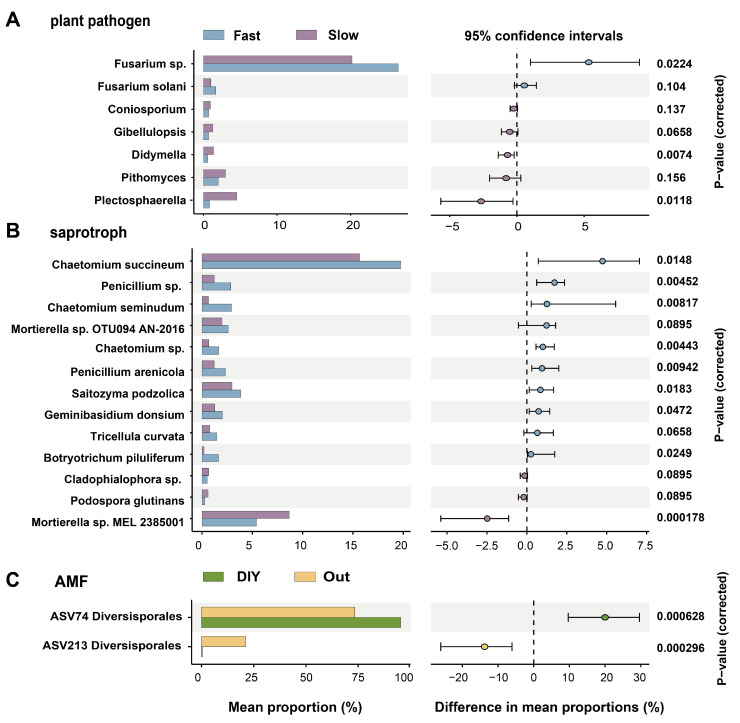
Statistical Analysis of Metagenomic Profiles (STAMP) analysis of fungal relative abundance associated with different root traits. The bar chart on the **left** illustrates the proportion of each amplicon sequence variant (ASV) associated with each root strategy. On the **right**, each dot represents the effect size (difference in relative abundance between groups), and the horizontal line indicates the 95% confidence interval (CI). The vertical dashed line indicates zero difference in mean proportions between groups. Statistical differences between groups were assessed using the non-parametric Wilcoxon rank-sum test, and *p*-values are shown as unadjusted values. AMF, arbuscular mycorrhizal fungi.

Finally, we conducted a STAMP analysis (Figure 6). The bar chart on the left illustrates the proportion of each ASV associated with each root strategy. The 95% confidence interval (CI) plot on the right further clarifies the differences between the two groups within each fungal community. The most abundant plant pathogenic genus identified was Fusarium, which significantly favored (*p* < 0.05) ‘fast’ roots (Figure 6A). Meanwhile, the two ascomycetes Plectosphaerella and Didymella significantly favored (*p* < 0.05) ‘slow’ roots (Figure 6A).

The predominant saprophyte was found to be Chaetomium succineum, which significantly favored (*p* < 0.05) ‘fast’ roots (Figure 6B). Other saprophytic ASVs significantly associated (*p* < 0.05) with the ‘fast’ strategy included Penicillium, Chaetomium, Saitozyma, and Botryotrichum piluliferum (Figure 6B). The only saprophyte found to significantly favor (*p* < 0.05) the ‘slow’ strategy was *Mortierella* sp. MEL 2385001 (Figure 6B).

Among AMF, two Diversisporales ASVs were identified. Among these, ASV213 was significantly associated (*p* < 0.05) with the ‘outsourcing’ strategy while ASV74 was significantly associated (*p* < 0.05) with the ‘DIY’ strategy (Figure 6C).

## 3. Discussion

### 3.1. Effects of Root System Strategies on Pathogenic Fungal Communities

We observed that green manures exhibiting ‘slow’ root traits or adopting ‘DIY’ strategies significantly increased soil pH (*p* < 0.05) (Figure 2B). As a key driver of plant pathogenic fungal community structure [30], soil pH not only shapes local soil environmental conditions but also regulates the expression of pathogenicity-related genes [22]. We also found that crops with ‘fast’ root traits amassed higher abundances of plant pathogenic fungi, (*p* < 0.05) (Figure 3), with the proliferation of Fusarium species serving as a representative example (Figure 6A). In addition, Mantel test analysis showed that soil pH was significantly associated with plant pathogenic fungi (*p* < 0.05) (Figure 2A). These findings align with prior studies showing that certain pathogens, such as Fusarium, are strongly favored under acidic conditions [31]. ‘Fast’ root traits are typically associated with high root nitrogen content, rapid root turnover, and low tissue density [15], which may be associated with greater inputs of labile root-derived carbon.

This shift in the root carbon profile creates conditions favorable for opportunistic pathogenic fungi (here referred to as r-selected pathogens), which are able to rapidly exploit nutrient-rich environments [32]. This may help explain the increased abundance of plant pathogenic fungi observed in this study (Figure 3). Even so, this ecological advantage may contribute to the increased relative abundance of pathogenic fungi and a corresponding decline in fungal diversity under the ‘fast’ root strategy (Figure 4). This pattern may reflect reduced niche differentiation under the ‘fast’ root strategy, wherein dominant species suppress community evenness. On the other hand, green manures employing a ‘DIY’ root strategy exhibited significantly reduced pathogen abundance (Figure 3) and diversity (Figure 4). This effect is likely attributable to the fact that these crops belonged to the Brassicaceae family, whose “biofumigant” effects are mainly associated with glucosinolates and their hydrolysis products, such as isothiocyanates, which can strongly inhibit soil-borne pathogens.

PCoA (Figure 5) showed that root trait differences were associated with distinct pathogenic fungal community structures, possibly related to variation in root-derived resource inputs [33]. Several possible explanations may underlie this pattern, including greater root-derived carbon inputs, frequent root renewal [33], and potentially reduced host defense capacity. Consequently, green manure with ‘fast’ root traits may suffer elevated disease risks even as they enhance productivity of subsequent crops [22]. Conversely, ‘slow’ root traits favored increased abundances of Plectosphaerella and Didymella (Figure 6A) [34], suggesting that these genera are adapted to stable, low-disturbance environments and highlighting the potential of ‘slow’-rooted green manures to suppress pathogens and support soil health [35].

### 3.2. Effects of Root System Strategies on Saprophytic Fungal Communities

Green manures with ‘slow’ root traits or adopting a ‘DIY’ strategy supported a higher abundance of saprotrophic fungi (Figure 3), yet were associated with lower Shannon diversities within this functional group (Figure 4). This discrepancy may reflect differences among saprotrophic taxa in their responses to fluctuations in carbon source dynamics and nutrient availability [36]. ‘Slow’ or ‘DIY’ root systems typically exhibit longer root lifespans and greater carbon-to-nitrogen (C/N) ratios, resulting in the continuous release of low-bioavailability carbon and thereby promoting SOC accumulation [37]. Stable carbon inputs also facilitate microbially mediated phosphorus transformation processes, consequently increasing TP content (Figure 2B) [38] and LAP activity (Figure 2A) [39]. Together, these processes establish a root ecosystem characterized by resource abundance and minimal environmental disturbance. Saprophytic fungi with more conservative resource-use strategies (here referred to as K-selected saprophytic fungi in a conceptual sense), such as *Mortierella* sp. MEL 2385001 (Figure 6B) are more likely to achieve ecological dominance in such an environment due to their highly efficient resource utilization and competitive stability [40]. However, colonization by K-selected fungi often results in significant exclusivity, which inhibits the coexistence of other saprophytic fungal taxa. This phenomenon may explain the observed decline in Shannon diversity accompanied by increased saprophytic fungal abundance [38].

In contrast, green manures exhibiting ‘fast’ root traits or ‘outsourcing’ strategies often release substantial amounts of readily decomposable root exudates in the short term, resulting in rapid nutrient enrichment [41]. This “explosive” resource input pattern favors the proliferation of r-selected saprophytes with short life cycles and fast growth rates [15] such as Chaetomium and Penicillium (Figure 6B). These microorganisms can rapidly respond to high carbon inputs and their short life cycles contribute to frequent community turnover, thereby increasing diversity [42]. However, the resultant community structures tend to be vulnerable to environmental fluctuations and demonstrate reduced ecological stability [43,44]. In addition, limited competitive inhibition allows for the coexistence of a greater diversity of saprophytic fungal taxa, thereby enhancing overall community diversity [36,45]. Finally, resource-unstable and less competitive ecosystems often experience reduced nutrient accumulation [46], which may account for the poorer soil properties and lower fungal abundances associated with ‘fast’ root traits or the ‘outsourcing’ strategy.

### 3.3. Effects of Root System Strategies on AMF Communities

AMF were significantly more abundant (Figure 3) and α-diverse (Figure 4) in association with crops characterized by ‘fast’ root traits or an ‘outsourcing’ strategy. This observation suggests that the high carbon input and rapid root turnover associated with ‘fast’ roots may facilitate a more diverse AMF assemblage [47]. Notably, both green manures with ‘fast’ roots and employing ‘outsourcing’ strategies were legumes, which are known to promote AMF symbioses. For example, the majority of nodulating species currently modeled are stable AMF fixers [48]. This may be explained by the fact that AMF tend to demand more nitrogen than phosphorus [11].

On the other hand, AMF community differentiation is primarily driven by variation within the order Diversisporales, although species-level annotations are limited. Members of Diversisporales frequently exhibit significant symbiotic plasticity and adaptability to nutrient availability [49], suggesting that their dominance may reflect functional specialization associated with different root trait strategies. The observed enrichment of AMF under the ‘outsourcing’ strategy suggests an intimate mutualistic relationship, potentially resulting from an increased reliance on the symbiotic uptake of nutrients from deeper soil layers [50]. From an applied perspective, leguminous green manures with an ‘outsourcing’ strategy may therefore be useful in rotation or intercropping systems for supporting beneficial AMF communities.

Compared to those utilizing an ‘outsourcing’ strategy, plants adopting the ‘DIY’ strategy demonstrate enhanced autonomous nutrient uptake and spatial exploration capabilities [51]. Such plants are less dependent on external symbionts for nutrient acquisition, which theoretically reduces the selective pressure to establish mutualistic relationships with arbuscular AMF. However, the two ‘DIY’ green manures utilized in this study are both members of Brassicaceae, which are not known to naturally form symbiotic relationships with AMF [52]. Furthermore, secondary metabolites produced by Brassicaceae plants, such as isothiocyanates, may inhibit the growth of AMF hyphae [53], further restricting their expansion within the ‘DIY’ community. Therefore, the low abundance and reduced α-diversity of the AMF community observed in association with ‘DIY’ green manures (Figure 3 and Figure 4) may not have resulted from any singular root strategy, but rather from the combined effects of the ‘DIY’ strategy, phylogenetic characteristics, and metabolic traits. This phenomenon underscores the importance of integrating root trait strategies with plant lineage and biochemical properties to comprehensively evaluate the regulatory mechanisms governing plant-microbe interactions and their impact on belowground community structures.

## 4. Materials and Methods

### 4.1. Study Area

This experiment was performed at the Dafangshan Experimental Field in Jinxian County, Jiangxi Province, China (116°35′13″ E, 28°10′57″ N). The site lies in a subtropical monsoon region with humid climatic conditions, where the mean annual temperature is 17.5 °C and the annual precipitation is approximately 1728 mm. The soil at the experimental site is derived from Quaternary red clay and is classified as red soil according to the Chinese soil classification system. Before the experiment began, the topsoil (0–20 cm) was acidic, with a pH of 4.68. The baseline soil properties were as follows: SOC, 12.31 g kg^−1^; TN, 1.11 g kg^−1^; TP, 0.55 g kg^−1^; AN, 0.55 g kg^−1^; and AP, 31.8 mg kg^−1^.

### 4.2. Experimental Design

Four green manures were utilized: hairy vetch (*Vicia villosa* Roth) (VV), rye (*Secale cereale* L.) (SC), radish (*Raphanus sativus* L.) (RS), and rapeseed (*Brassica napus* L.) (BN). These species were chosen because they represent contrasting plant functional types and were expected to differ in root trait strategies, thereby providing a suitable basis for examining relationships between root economics and soil fungal communities. Each treatment was arranged in a completely randomized block design, with each plot measuring 30 m^2^ and consisting of six replicate subplots. Each subplot was treated as an independent experimental unit in the statistical analyses. The green manures were sown by broadcasting in late October 2022. In accordance with local agricultural practices, the seeding rates were 40, 20, 30, and 10 kg ha^−1^ for hairy vetch, rye, radish, and rapeseed, respectively. No irrigation or fertilization was applied during the green manuring period. All green manures were mechanically incorporated at flowering (early April 2023) to a depth of 10 to 20 cm. Sweet potatoes (*Ipomoea batatas* L.) were subsequently transplanted in early May, with a ridge spacing of 1 m, a plant spacing of 20 cm, and a planting density of 50,000 plants ha^−1^.

### 4.3. Root Trait Measurements and Soil Sampling

Green manure root samples were collected during the flowering period (mid-March 2023). Five representative plants were selected from each plot, excavated with the root systems intact, placed in sample bags, and transported to the laboratory. After gently washing away adhering soil with tap water, root samples consisting predominantly of fine roots were used for scanning and trait measurements. Root length (RL, cm), root volume (RV, cm^3^), and root diameter (RD, mm) were measured using a root scanner (EPSON LA2400, Seiko Epson, Suwa, Japan) according to the method of Hennecke et al. [6]. Within each plot, the five plants were processed separately and treated as biological replicates for root trait analysis. The scanned roots were subsequently weighed, dried (at 70 °C for 48 h), and weighed again. Specific root length (SRL) was calculated as the ratio of root length to dry mass, and root tissue density (RTD) was calculated as the ratio of root dry mass to root volume [5,6]. Finally, root samples were manually ground and their nitrogen (RN) and carbon (RC) contents were measured using an elemental analyzer (Elementar Vario ELII, Hanau, Germany).

At the seedling stage (late May 2023), five soil cores (diameter 2.0 cm) were collected from five locations within each sweet potato plot at a depth of 0–20 cm. The cores were thoroughly mixed to form a composite sample (500 g), resulting in a total of 24 samples (4 treatments × 6 replicates). The soil samples were placed in an insulated box with ice packs and immediately returned to the laboratory. Impurities were removed in the laboratory and one portion of each sample was air-dried after sieving (2 mm) for physicochemical assessment while another portion was stored at −20 °C for microbial community analysis.

### 4.4. Measurement of Soil Physicochemical Properties

Soil physicochemical properties were analyzed according to the method of Jiang et al. [54]. Soil pH was evaluated via potentiometry (water:soil = 2.5:1). Soil organic carbon (SOC) was quantified using the potassium dichromate oxidation technique. Total nitrogen (TN) was quantified using the Kjeldahl approach, total phosphorus (TP) was assessed via sodium carbonate fusion, soil available phosphorus (AP) was evaluated using sodium bicarbonate extraction method combined with molybdenum-antimony resistance colorimetry, and soil available nitrogen (AN) was determined via alkaline hydrolysis. The activities of β-1,4-glucosidase (BG), β-xylosidase (XYL), β-D-cellobiohydrolase (CB), β-1,4-N-acetylglucosaminidase (NAG), leucine aminopeptidase (LAP), and acid phosphatase (ACP) were measured using a fluorometric assay [55]. Soil physicochemical variables were further included as explanatory variables in the multivariate analyses examining relationships among soil properties, fungal functional groups, and root trait strategies.

### 4.5. Sequencing and Bioinformatics Analysis

DNA was extracted from each 0.5 g soil sample using the FastDNA^®^ SPIN Kit for Soil (MP Biomedicals, Solon, OH, USA) according to the manufacturer’s instructions. The ITS1F (5′-CTTGGTCATTTAGAGGAAGTAA-3′) and ITS2R (5′-GCTGCGTTCTTCATCGATGC-3′) internal transcribed spacer (ITS) primer pair was employed to analyze fungal community structure [56]. PCR amplification of the fungal ITS region was performed under the following conditions: initial denaturation at 94 °C for 3 min; 27 cycles of denaturation at 95 °C for 30 s, annealing at 55 °C for 30 s, and extension at 72 °C for 45 s; followed by a final extension at 72 °C for 10 min. The resulting PCR products were subjected to paired-end sequencing (2 × 250 bp) on the Illumina MiSeq PE250 platform (Shanghai LingEn Biotechnology Co., Ltd., Shanghai, China).

### 4.6. Sequencing Data Processing

The amplified sequences were analyzed using the QIIME2 analysis pipeline. Briefly, the sequences were filtered and denoised with ‘cutadapt’ and ‘Demux’ [57] to ensure the accuracy of subsequent analyses. The quality-controlled sequences were merged into paired-end reads utilizing the DADA2 algorithm in R (v.4.5). Following dereplication, amplicon sequence variants (ASVs) were inferred using DADA2 at single-nucleotide resolution. Following ASV generation, functional group classification (saprotrophs, plant pathogens, and AMF) was conducted in conjunction with the FungalTraits database [58].

### 4.7. Data Analysis

All statistical analyses were performed in R Studio (v.4.5; R Core Team, 2025). The ‘ggplot2’ package was employed to create figures. For the differential abundance and richness analysis among groups, statistically significant differences were evaluated using the non-parametric Kruskal–Wallis test to assess intergroup variation. If the Kruskal–Wallis test results indicated a statistically significant difference (*p* < 0.05), pairwise comparisons were subsequently conducted using the Wilcoxon rank sum test. In the present analysis, pairwise *p*-values were reported as unadjusted values. The Kruskal–Wallis test was implemented using the ‘kruskal.test’ function, while pairwise Wilcoxon tests were performed using the ‘ggpubr’ package.

Principal component analysis (PCA) was performed on RN, RD, SRL, and RTD using the ‘vegan’ package to calculate the proportion of variance explained by each principal component (PC). Correlations between soil fungal functional groups and environmental variables were analyzed using a Mantel test implemented with the ‘ggcor’ package. Relationships between environmental factors (pH, SOC, TP, β-D-cellobiohydrolase (CB) and leucine aminopeptidase (LAP)), fungal groups, and root trait strategies were also analyzed. The same method was employed to analyze the relationship between root traits and fungal abundance and diversity (Shannon index). β-diversity was assessed using principal coordinates analysis (PCoA) based on the unweighted UniFrac distance matrix. Finally, the Statistical Analysis of Metagenomic Profiles (STAMP) analysis pipeline was used to conduct intergroup analysis of microbial relative abundance. The non-parametric Wilcoxon rank-sum test was employed to assess differences in taxon abundance among treatments, combined with effect size measures (such as differences) and 95% confidence intervals to evaluate the actual impact.

## 5. Conclusions

In this study, we used Root Economics Space (RES) theory to explore the associations between green manure root functional strategies and soil fungal guild structure. Overall, different root strategies were associated with distinct patterns in fungal guild composition, abundance, and diversity, possibly related to differences in soil pH, root-derived resource inputs, and microbial niche conditions. Green manures exhibiting ‘slow’ roots or utilizing ‘DIY’ strategies were associated with lower relative abundance of plant pathogenic fungi and higher relative abundance of saprotrophic fungi. In contrast, ‘fast’ roots and ‘outsourcing’ strategies were associated with higher relative abundance of plant pathogens and AMF. These findings suggest that different root strategies may influence fungal abundance, diversity, and community structure in distinct ways. However, these strategies may also promote disease and ecological instability. Therefore, optimizing the selection and configuration of green manure species based on root traits may provide a useful approach for regulating soil microecology, reducing soil-borne disease risk, and improving the sustainability of agricultural systems.

## Data Availability

The datasets for this study can be found in SRA of the NCBI under accession number PRJNA1308463.
